# Elaboration of a nomogram to predict non sentinel node status in breast cancer patients with positive sentinel node, intra-operatively assessed with one step nucleic acid amplification method

**DOI:** 10.1186/s13046-015-0246-2

**Published:** 2015-11-04

**Authors:** F. Di Filippo, D. Giannarelli, C. Bouteille, L. Bernet, R. Cano, G. Cunnick, A. Sapino

**Affiliations:** Regina Elena National Cancer Institute, Via Elio Chianesi 53, 00134 Rome, Italy; Clinique Mutualiste, Saint Etienne, France; Hospital de Xàtiva, Valencia, Spain; Hospital de Alzira, Valencia, Spain; Wycombe General Hospital, Buckinghamshire, England; Istituto di Candiolo - IRCCS, Fpo-Ircc., Turin, Italy; Dept of Medical Sciences - University of Turin, Turin, Italy

**Keywords:** Nomogram, Non Sentinel Node status, OSNA method, CK19 mRNA number copies

## Abstract

**Backgrounds:**

Tumor-positive sentinel node(SLN) biopsy results in a risk of nonsentinel node metastases in case of micro and macro metastases ranging from 20 to 50 %, respectively. Therefore, most patients underwent unnecessary axillary lymph node dissections.

Thus, the development of a mathematical model for predicting patient-specific risk of non sentinel node(NSLN) metastases is strongly warranted.

**Methods:**

The following parameters were recorded:

- Clinical: hospital, age, medical record number
- Bio-pathological: tumor (T) size, grading (G), multifocality, histological type, LVI, ER-PR status, HER-2, ki67, molecular classification (luminal A, luminal B, HER2 like, triple negative)
- Sentinel and nonsentinel lymph node related: number of removed SLNs, number of positive and negative SLNs, copy number of positive sentinel nodes, ratio: number of positive SLNs to number of removed SLNs, number of removed and number of positive nodes after ALND. A total of 2460 patients have been included in the database.

All the patients have been provided by the authors of this paper.

**Results:**

Multivariate logistic regression analysis demonstrated that only the number of a CK19 mRNA copies (*p* < 0.0001), T size (*p* < 0.0001) and LVI (*p* < 0.0001) were associated with NSN metastases.

The discrimination of the model, quantified with the area under the receiver operating characteristics curve, was 0.71 (95 %, C.I. 0.69–0.73), thus confirming a good level of reliability.

**Conclusions:**

The nomogram may be employed by the surgeon as a decision making tool on whether to perform an intraoperative axillary lymph node dissection on breast cancer patients with SLN positive.

The large population employed and the standardized method of measuring the value of CK19 mRNA copies are appropiate prerequisites for a reliable nomogram.

## Background

Breast cancer is one of the most frequent neoplasm in women, generally treated with quadrantectomy or mastectomy [1].

Sentinel lymph node (SLN) biopsy is a highly accurate predictor of overall axillary status and has become the standard axillary staging method for the last 15 years in breast cancer (BC) patients who are confirmed clinically negative for lymph node metastases [2, 3]. In the case of negative SLN, patients can safely avoid axillary lymph node dissection (ALND), thus preventing associated morbidity [4]. However, approximately 50–70 % of patients with positive SLN have no additional positive nodes, suggesting that it may be possible to avoid ALND in selected patients [5, 6]. Taking these considerations into account, an accurate estimate of the likelihood of additional node metastases may be of paramount importance when deciding further treatment. At the present time, the intraoperative decision on, whether to perform ALND or not, is often only based on the positivity of the SLN. In order to assess the SLN status more rapidly, a semi-automated molecular method called the one step nucleic acid amplification (OSNA) assay has recently been made available [7–11]. On the basis of these considerations, the European OSNA Committee decided to develop a new nomogram able to predict the non sentinel node (NSN) status, including the number of CK 19 mRNA copies as the most powerful parameter. The aim of the study was to report the results of the retrospective phase of the Nomogram Project, as the validation phase is ongoing.

## Methods

The European OSNA Users Committee decided to develop a Nomogram Project with the following aims:

- To create new predictive factors for NSN positivity (copy number of CK19 mRNA) as well assessing the conventional histopathological parameters.
- To develop a user-friendly nomogram to predict NSN positivity based on the CK19 mRNA copy determined by OSNA assay tested in large patient population.

Our study population only included cases that fulfilled the following criteria: primary invasive cT1-3 BC with clinically and radiological (preoperative sonogram) negative axilla; no prior systemic treatment, or axillary surgery; successful SLN biopsy in which metastatic disease was identified by OSNA; and ALND with at least 10 nodes examined.

The following parameters were recorded:

- Clinical: hospital, age, medical record number
- Bio-pathological: tumor size, grading, multifocality, histological type, LVI, ER-PR status, HER-2, ki67, molecular classification (luminal A, luminal B, HER2 like, triple negative)
- SLN and NSN related: number of removed SLNs, number of positive and negative SLNs, copy number of positive SLNs, ratio: number of positive SLNs to number of removed SLNs, number of removed and number of positive nodes after ALND. A total of 2460 patients have been included in the database.

The biopathological parameters included in the database are shown in Tables 1 and 2.

**Table 1:** Clinicopathologic characteristic of patients

Characteristics	N of patients	Percent
HYSTOLOGY		
IDC	2140	87.0
ILC	320	13.0
GRADING		
G1	389	15.8
G2	1395	56.7
G3	676	27.5
ER		
pos	2213	90.0
neg	247	10.0
PgR		
pos	1984	80.6
neg	476	19.4
HER2		
pos	162	6.5
neg	2298	93.5
Ki67		
low	1271	51.7
high	1189	48.3
T		
≤12	638	25.9
≥13–18	676	27.5
≥19–25	642	26.1
>25	504	20.5
TYPE^a^		
multiple	378	15.4
single	2082	84.6
LVI		
no	1397	56.8
yes	1063	43.2
Luminal A	1185	48.1
Luminal B	982	39.9
HER2-like	46	1.9
Triple Negative	247	10.1

^a^multiple stands for multifocality

**Table 2:** Characteristics of sentinel node and non sentinel node

	Number	Percent
N° of SLN Examined		
1	1273	51.7
2	801	32.6
3	277	11.3
4	88	3.5
5	17	0.7
6	4	0.2
N° of positive SLN		
1	1765	71.8
2	527	21.4
3	136	5.5
4	29	1.2
5	3	0.1
SLN Micrometastases	977	39.7
SLN Macrometastases	1483	60.3
Median (range)	14 (2–47)	
N° of NSLNs removed mean	14.8	
N° of positive NSLNs	829	33.7
Median (range)	2 (1–30)	
Mean	3.2	
N Ratio: N° SLN / N° SLN removed		
<0.25	23	0.9
0.25–0.50	491	20.0
0.50–1.00	111	4.5
1 .00	1835	74.6
N° of Copies (Highest copy number)		
≤1500	615	25.0
>1500–12,000	636	25.9
>12,000–111,000	608	24.7
>111,000	601	24.4
N° of Copies (Total Tumor Load)		
≤1500	607	24.7
>1500–12,000	625	25.4
>12,000–111,000	612	24.9
>111,000	616	25.0

*multiple stands for multifocality

Twenty-two European centers contributed to the enrollment of patients between January 2008 and February 2013; 2460 patients made up the body of nomogram.

### Sentinel Lymph Node sampling method

SLNs were identified using technetium 99 m- labeled, nanosized, human serum albumin colloids. To avoid any contamination during tumor manipulation, SLNs were surgically excised before breast surgery and sent on ice to the Pathology Department.

Each SLN was weighed and measured. SLNs weighing less than 50 mg were excluded from the study. SLNs weighing more than 600 mg were cut in two or more pieces and processed as separate nodes.

### One Step Nucleic Acid Amplification

The OSNA assay was performed according to the manufacturer’s instructions (Sysmex, Kobe, Japan). In short, the SLN was homogenized in 4 ml of the LINORHAG homogenizing buffer (Sysmex) on ice. A small aliquot was used for automated real- time amplification of CK19 mRNA via reverse transcription loop-mediated isothermal amplification (RT-LAMP) with the ready-to use LYNOAMP reagent kit (Sysmex) on the RD-100i (Sysmex).

It was possible to analyze up to 4 SLNs in one run.

The degree of amplification was detected via a by-product of the reaction, i.e. magnesium-pyrophosphate. After use, the excess lysate was stored at minus 80 °C. A lysate with CK19 mRNA copy number/μl less than 250 (a) was regarded as negative (score−); from 250 to 5000 (b) as positive (score +), and greater than 5000 (c) (score ++). The OSNA results were immediately communicated by telephone to the surgeon within 30–40 min. For statistical analysis, in case of two or more SLNs, the SLN with the greatest CK19 mRNA copies was chosen.

When there was a positive OSNA result, both for micrometastases (+) and macrometastases (++) the patients underwent an immediate ALND. ITC are not detected by OSNA method. This is not a limitation because patients with positive SN for ITC are no longer submitted to ALND.

Axillary NSNs were routinely examined by H&E.

### Statistical analyses

The outcome of our nomogram was the presence of positive nodes in the axillary dissections following OSNA evaluation in the population defined above. We identified a list of potential covariates that may predict this outcome, thus the endpoint was a binary outcome (presence versus absence of at least one positive node other than SLN) and the association with the covariates was analyzed using a logistic linear model. Continuous variables (number of CK19 mRNA copies and T size expressed in mm) were categorized using quartiles. We investigated the role of each variable and estimated the Odds Ratio along with the 95 % confidence interval; independent factors were then identified using a stepwise forward likelihood ratio method.

Discrimination ability was assessed by ROC analysis and predictive accuracy was measured by the AUC reported with its 95 % confidence interval. Calibration was evaluated by reviewing the plot of predicted probabilities versus the actual probabilities. Well calibrated models have a linear relationship with a slope of 1 and an intercept of 0. Thus, a linear regression coefficient between predicted and observed values was estimated.

The resulting model will be validated in a prospective series. All the analyses were performed using IBM SPSS version n. 20 [12].

### Ethical consideration

The patient data was anonymously gathered retrospectively with no influence on patient therapy. The Nomogram project was approved by Ethical Committee of each participating institution.

## Results

Table 1 shows the clinical and bio-pathological characteristics of the patients.

The mean and median ages were 56 and 54, respectively ranging between 22 and 90 years.

The vast majority of the patients were affected with infiltrating ductal carcinoma (87.0 %). Most of them had an intermediate (56.7 %) or high grade tumors (27.5 %). Both Estrogen (ER) and Progesterone (PgR) receptors were positive in 90 and 80.6 %, respectively, whereas HER2 was positive only in 6.5 % of the patients. Ki67 was high in 48.3 % and LVI was present in 43.2 % of the patients. These parameters represent the new molecular classifications of breast cancer that allow not only to identify patients at a higher risk of relapse but may also guide postoperative therapies [13, 14].

Tumor size was divided in quartiles, the cut-offs being 12, 18 and 25 mm.

The mean and median tumor sizes were 20.3 and 18 mm, respectively, ranging between 0.8 and 50 mm.

The SLNs and NSLNs characteristics are reported in Table 2. Patients with micro or macrometastases in the SLNs were 977 (39.7 %) and 1483 (60.3 %), respectively.

The median number of examined and positive SLNs was 1, one SLN was examined in 1273 patients (51.7 %); 2 in 801 (32.6 %); 3 in 277 (11.3 %); 4 in 88 (3.5 %); 5 in 17 (0.7 %); 6 in 4 (0.2 %).

1765 patients (71.8 %) had only 1 positive SLN, 527 (21.4 %) had two, 136 (5.5 %) had three, 29 (1.2 %) had four and 3 (0.1 %) had five. NSLNs were positive in 829 patients (33.7 %), found both in patients with SLN micrometastases (22 %) or macrometastases (78 %). The median and mean number of positive NSLNs were 2 and 3.2, respectively, ranging between 1 and 30.

The mean and median number of NSLNs removed were 14.8 and 14, respectively. The number of CK19 mRNA copies was divided in quartiles in order to obtained a better stratification of the patients.

In Table 3 the results of univariate and multivariate analyses are reported.

**Table 3:** Results of Univariate and Multivariate Analysis

Univariate analysis			Multivariate analysis	
	OR (95 % c.i.)	*P*	OR (95 % c.i.)	*P*
HYSTOLOGY		0.44		
CDI vs CLI	0.90 (0.69–1.17)			
GRADING		0.001		
G2 vs G1	1.45 (1.12–1.87)	0.005		
G3 vs G1	1.74 (1.32–2.31)	<0.0001		
ER		0.27		
Pos vs neg	1.19 (0.87–1.61)			
PgR		0.09		
Pos vs neg	1.22 (0.97–1.53)			
HER2		0.11		
Pos vs neg	0.73 (0.50–1.07)			
Ki67		0.01		
High vs low	1.29 (1.06–1.56)			
T		<0.0001		<0.0001
13–18 vs <12	1.31 (1.02–1.67)	0.03	1.10 (0.85–1.43)	0.45
19–25 vs <12	2.04 (1.60–2.60)	<0.0001	1.73 (1.34–2.23)	<0.0001
>25 vs <12	2.70 (2.10–3.48)	<0.0001	2.22 (1.70–2.90)	<0.0001
TYPE*		0.001		
Multiple vs single	1.55 (1.19–2.02)			
LVI		<0.0001	n.c.	
Yes vs No	2.88 (2.37–3.49)			
COPIES		<0.0001		
Macro (>5000) vs Micro (<=5000)	3.57 (2.95–4.32)			
COPIE		<0.0001		<0.0001
1500–12,000 vs <1500	2.15 (1.62–2.85)	<0.0001	2.15 (1.60–2.87)	<0.0001
12,000–111,000 vs <1500	4.26 (3.24–5.62)	<0.0001	4.14 (3.12–5.49)	<0.0001
>111,000 vs <1500	7.18 (5.46–9.45)	<0.0001	6.86 (5.18–9.01)	<0.0001
MOLECULAR SUBTYPE		0.05		
Luminal A vs triple neg	1.09 (0.76–1.56)	0.64		
Luminal B vs triple neg	1.39 (0.97–2.01)	0.07		
Her2 vs triple neg	0.76 (0.32–1.80)	0.54		

Logistic regression model showed that grading (*p* < 0.0001), Ki67 (*p* < 0.01), type (*p* < 0.001), T size (*p* < 0.0001), size of metastatic foci (micro or macrometastases) in the SLN (*p* < 0.0001) and number of CK19 mRNA copies (*p* < 0.0001) were associated with positive NSN status.

Multivariate logistic regression using a Cox model analysis demonstrated that only the number of CK19 mRNA copies (*p* < 0.0001), T size (*p* < 0.0001) and LVI (*p* < 0.0001) maintained their independent value, and therefore they would be useful to construct the nomogram. We wanted to build a nomogram that the surgeon could utilize during the operation, therefore LVI was excluded because its unreliability was assessed in preoperative core biopsy only.

According to the clinical-pathological characteristics of our patients, the predicted probability of NSN positivity ranges between 9.9 and 64.3 %.

One of the main aim of nomogram is to identify the subgroup of patients with a low risk of NSN involvement [15]. When calculating the number of patients with a probability of metastatic NSN ≤ 10 %, predicted by the nomogram, 212 patients could be identified in this subset. Among these patients, 196 patients were truly negative and only 16 (7.5 %) were falsely negative. It must be emphasized that the percentage of 7.5 % is, perfectly superimposable to false negative rate of SLN biopsy normally quoted at 5–10 % [16, 17].

The model showed a sensitivity of 98.1 % and a specificity of 19 %, whilst the positive and negative predict values were 36.2 and 92.5 %, respectively.

**Fig. 1:**
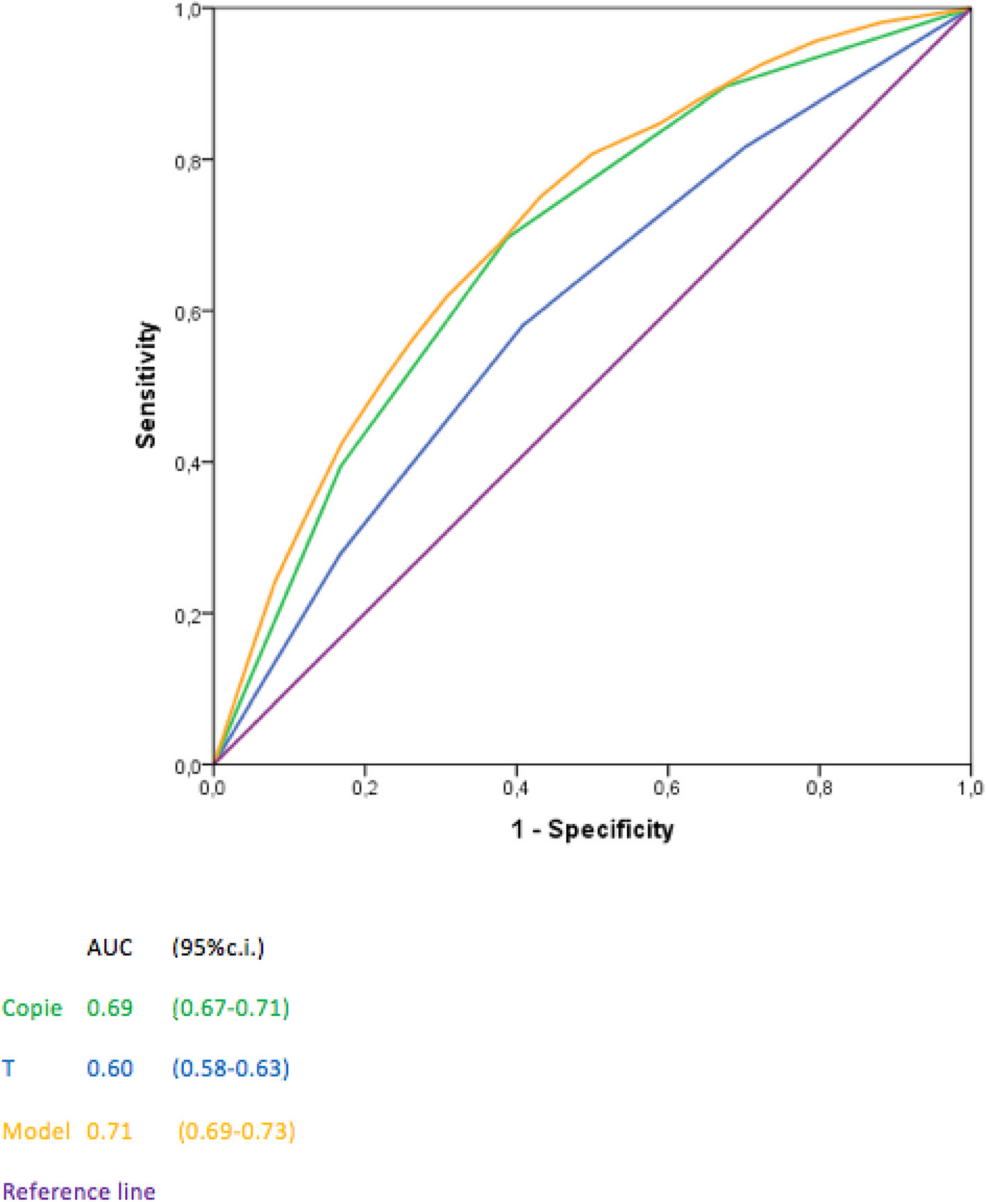
ROC curves of number of CK19 mRNA, T size (quartiles) and the model containing these two variables

The discrimination of the model, quantified with the area under receiver operating characteristic (ROC) curve was 0.71 (95 % c.i. 0.69–0.73) showing a good level of discrimination (Fig. 1).

**Fig. 2:**
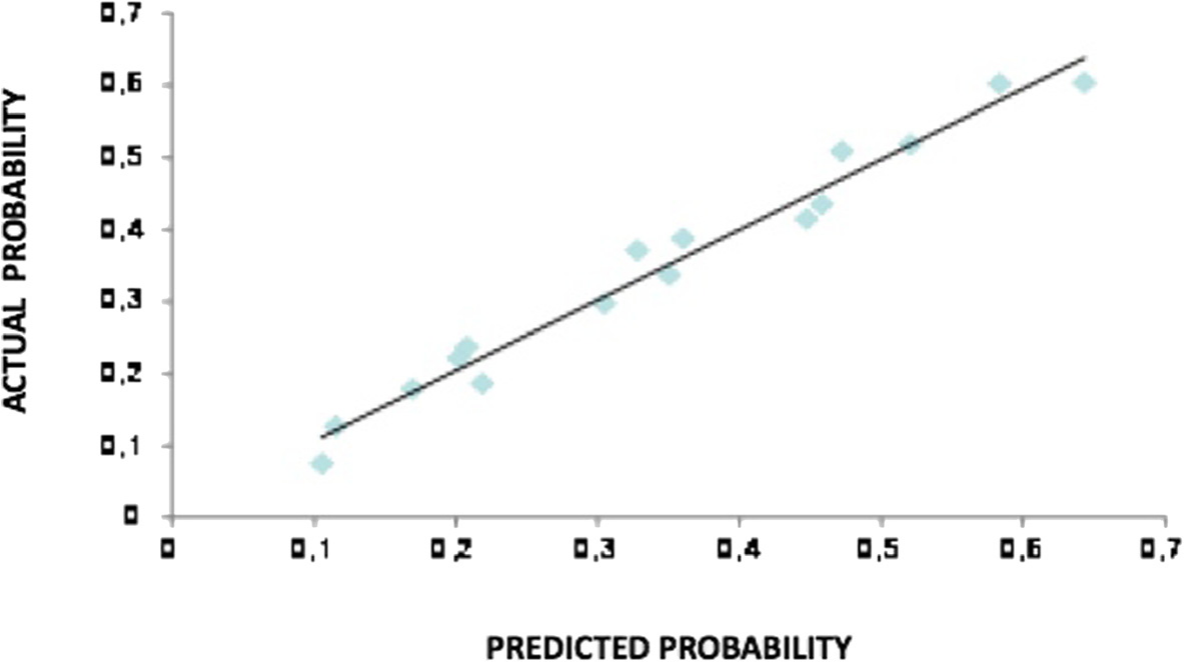
The model performs well and correctly both at low and in high risk cases as shown in calibration plot. The linear regression model has a slope of 0.98 (95 %, C.I. 0.89–1.07) and a constant of 0.01 between predicted and actual probabilities (95 %, C.I. 0.03–0.04)

The model performed well and correctly both in low and in high risk cases as shown in the calibration plot. The linear regression model between has a slope of 0.98 (95 % c.i. 0.89–1.07) and a constant of 0.01 (95 % c.i. -0.03–0.04) between predicted and actual probability (Fig. 2).

**Fig. 3:**
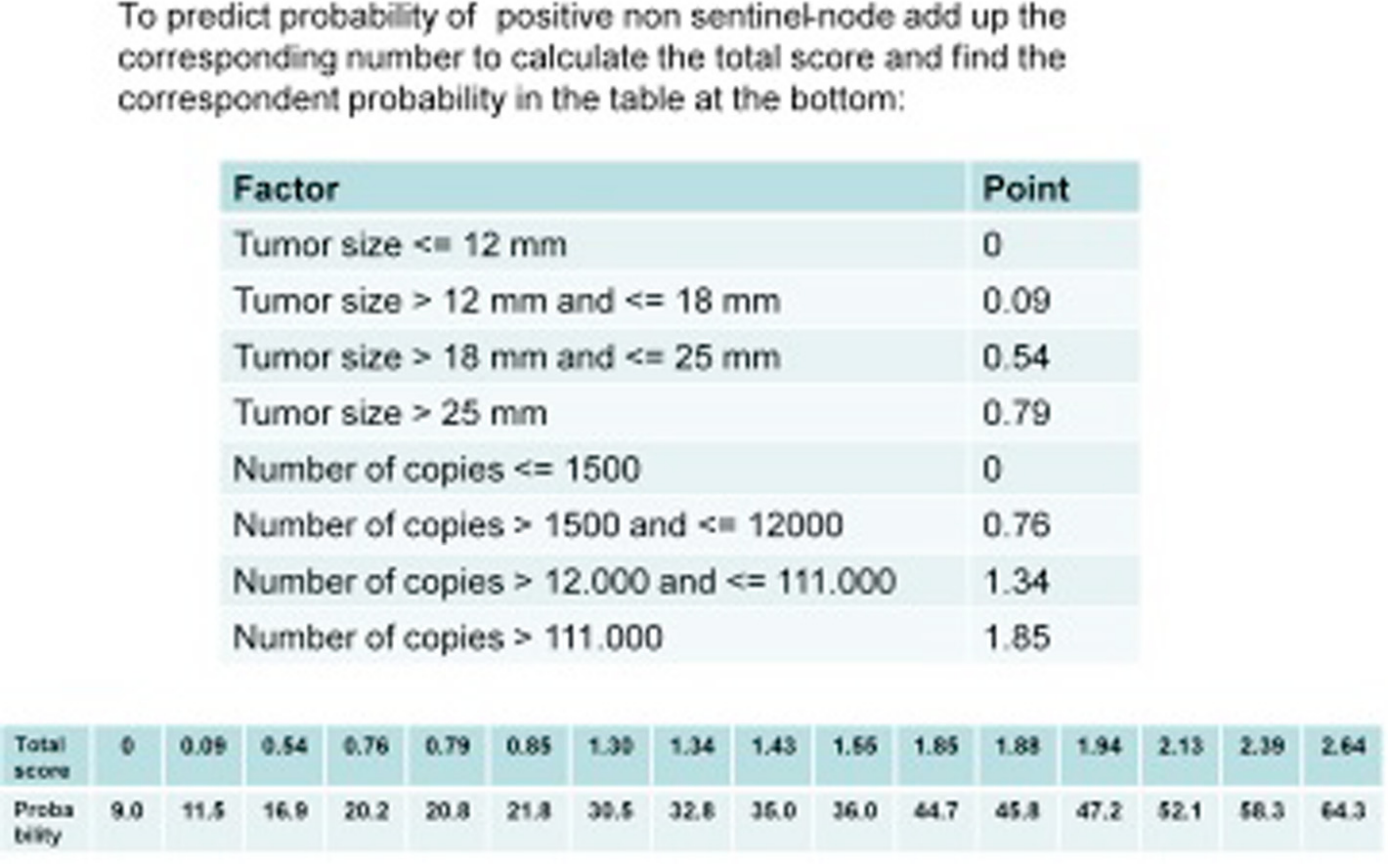
Nomogram to calculate the risk percentage of NSN positivity. The score of each of the 2 variables are summed and reported on the total score raw, immediately below the percentage of NSN positivity is identified

The significant variables were then incorporated into a nomogram to predict NSN status (Fig. 3).

## Discussion

It is well known that SLN micro- and macrometastases are associated with mean NSN positivity rate of 20 and 50 %, respectively. Consequently, the dilemma for surgeons still persists in how to avoid unnecessary ALND and how to identify patients at low risk of NSN positivity.

Recently, ASCO guidelines for SLNB and ALND have been published, indicating the patients with micro and macrometastases (only those that meet Giuliano criteria) may avoid ALND, but many controversies still remain [18]. The Z0011 Trial that randomized patients to observation or ALND after positive SLN, failed to reach its target accrual. However, the analysis restricted to only 856 patients seemed to demonstrate no differences in disease-free and overall survival between the two groups [19]. But the limited number of patients together with the fact that 97 % of patients received systemic chemotherapy and axillary radiotherapy (89 %) obscures the reliability of the results. Moreover, the results of this study cannot be applied to subsets of patients like young women (<50 years), patients undergoing mastectomy, patients with lobular carcinoma or hormone receptor negative tumours or HER2 positive tumours, due to being underrepresented in Z0011 study. Finally in 2012, the CAGS/ACS Based Review in Surgery Committee examined the Z0011 trial because its results might be “practice changing”. A great deal of biases were found and the final conclusion was: “Should the results of Z0011 change practice? Owing to its methodological limitations, if we had to depend on Z0011 alone, the standard of care following positive sentinel node is still an ALND” [20]. The same conclusion was reached by a German, Austrian and Swiss (D, A, CH) consensus panel in 2013 [21].

Recently, the prospective randomized IBCSG23-01 trial has been published [22]. Only patients with micrometastases were randomized to either ALND or no further treatment in patients with positive SLN.

The results of this study demonstrated no differences between the two arms both in terms of disease-free and of overall survival.

Some challenges, however, still exist regarding this study. Patients accrual stopped prematurely and only 933 out of 1960 patients were enrolled, therefore the study was underpowered.

The patient population had a very good prognosis. In fact, sentinel tumor size ≤ 1 mm was present in 69 % of the patients. As a result, the incidence of additional positive NSN in axillary dissection group was 13 %, very similar to that found in case of ITCs metastases in SLN. This is also because the author included ITCs in the group of micrometastases. A clear correlation between the size of micrometastases (less or greater than 1 mm) and positive NSNs was clearly demonstrated by Rahusen and Viale [23, 24]. Their results confirm that the presence in the study of 69 % of patients with SLN micrometastases ≤ 1 mm is a great bias. The median 5-years follow-up is too short to assess the real incidence of axillary recurrence in this study. In NSABP-B6, 20 % of nodal recurrences after lumpectomy and 24 % of nodal recurrences after lymphadenectomy and radiotherapy occurred after 5 years [25]. These data are very consistent with the Dutch Mirror study that demonstrated an increased recurrence rate in patients with micrometastases in the SLN not undergoing ALND [26].

Taking into account these considerations, accurate estimates of the likelihood of additional nodal metastases may be of paramount importance in the decision making process regarding further treatment. Nomograms might be valid prediction tools for surgeons to select patients that significantly benefit from an ALND after positive SLN.

The European OSNA Users Committee decided to collect a high number of patients use whose SLNs were all assessed with OSNA. In fact, our nomogram elaborated 2460 patients.

To the best of our knowledge, this is the largest prospective series in which patients with micrometastases (+) and macrometastases (++), detected by the OSNA assay, underwent immediated ALND.

Table 3 shows that multivariate regression Cox analysis selected the number of CK19 mRNA copies, T size and LVI as independent prognostic factors. LVI was not employed to develop the nomogram for its unreliability to assess this parameter with only a preoperative core biopsy. OSNA classifies SLN micrometastases when the number of CK19 mRNA copies ranges between 250 and 5000; whereas the number of copies > 5000 identifies macrometastases.

Therefore, for both micro and macro-metastases there is a wide range of number of copies, and according to the number of copies, patients may have different probabilities of positive NSN [11].

In our nomogram, we used the number of copies as a continuous variable divided in quartiles in order to have approximately the same number of patients in each subgroup with a more reliable patient distribution. The use of SLN size as a continuous variable improved the capacity of nomogram to accurately predict the NSN involvement in patients with positive SLN, as demonstrated by Mittendorf [27].

The availability of a molecular method which provides less subjective and quantitative results may be a useful tool in this context. We also evaluated T size as a continuous variable dividing patients in quartiles to obtain a better distribution.

The association of tumor size with the likelihood of NSN metastasis has been documented in numerous studies [15, 28–36].

It is readily apparent that the two parameters chosen for our nomogram reflect the greatest probability to identify patients with the NSN metastasis.

There are several parameters that permits to evaluate the reliability of a nomogram.

### Discrimination

In our nomogram the AUC is 0.71 (0.69 – 0.73) which is considered a good value of discrimination and is consistent with the best nomograms published so far [37–43].

### Calibration

Calibration determines the distance between predicted outcome and actual outcome, and has a higher clinical significance than discrimination [27, 37].

In our nomogram, the mean difference between predicted and calibrated probabilities was 2.3 %, with a maximum difference of 4.0 % (Fig. 2). This information is of clinical utility because it gives the opportunity for the clinicians to inform the patient regarding both the predicted probability of NSN metastases and the range of probability.

### False negative rate

It must be emphasized that our nomogram was able to identify 212 patients with a risk of NSN metastases ≤ 10 %. Only 16 patients were FN (i. e. presence of metastases) in NSLNs. Therefore, the FN rate is 7.5 %, thus confirming the validity of the nomogram to identify patients that may omit ALND. This makes both the surgeon comfortable in the decision making process as well as the patient to accept this decision.

The multivariate logistic regression analysis showed that the number of copies is an independent predictor of metastatic NSLNs after adjusting for T size (Fig. 1).

It must be stressed that the number of copies is assessed intra-operatively. Therefore, the surgeon may decide on whether to perform an ALND or not in the same operation, avoiding the psychological impact of a second operation.

However the result of this nomogram are currently under external validation to test reproducibility of the model on a large series of independent prospective data. This method remains the best choice in validating the proposed nomogram and in making a reliable instrument available.

## Conclusions

In conclusion, there is still ongoing discussion and debate among breast cancer surgeons regarding the need to perform completion ALND in patients with positive SLN. This nomogram incorporating SLN tumour burden, defined by OSNA method, is another tool that can be utilized by surgeons to more effectively counsel individual patients, thereby personalizing the surgical treatment of breast cancer and using this information intra-operatively.

## Abbreviations

ALND: Axillary lymph node dissection
BC: Breast cancer
ER: Estrogen receptor
ITC: Isolated tumor cell
LVI: Lymphovascular invasion
NSN: Non sentinel node
OSNA: One step nucleic acid amplification
PgR: Progesterone receptor
RT-LAMP: Reverse transcription loop-mediated isothermal amplification
ROC: Operating characteristics curve
SLN: Sentinel lymphonode
